# Adsorption of Cadmium and Lead Capacity and Environmental Stability of Magnesium-Modified High-Sulfur Hydrochar: Greenly Utilizing Chicken Feather

**DOI:** 10.3390/toxics12050356

**Published:** 2024-05-11

**Authors:** Weiqi Deng, Xubin Kuang, Zhaoxin Xu, Deyun Li, Yongtao Li, Yulong Zhang

**Affiliations:** 1 Key Laboratory of Arable Land Conservation (South China), Ministry of Agriculture, College of Natural Resources and Environment, South China Agricultural University, Guangzhou 510642, China; deng79141@163.com (W.D.); 15918625625@163.com (X.K.); xuzhaoxin0643@163.com (Z.X.); yongtao@scau.edu.cn (Y.L.); 2 College of Natural Resources and Environment, Joint Institute for Environmental Research & Education, South China Agricultural University, Guangzhou 510642, China; 3 WENS Foodstuff Group Co., Ltd., Yunfu 527400, China; 4 School of Environmental Science and Engineering, Shaanxi University of Science & Technology, Xi’an 710021, China; deyun_94@163.com

**Keywords:** heavy metal, adsorption mechanism, keratin, ^13^C NMR, water-soluble fertilizer

## Abstract

Chicken feathers represent a viable material for producing biochar adsorbents. Traditional slow pyrolysis methods often result in sulfur element losses from chicken feathers, whereas hydrothermal reactions generate substantial amounts of nutrient-rich hydrothermal liquor. Magnesium-modified high-sulfur hydrochar MWF was synthesized through magnesium modification, achieving a S content of 3.68%. The maximum equilibrium adsorption amounts of MWF for Cd^2+^ and Pb^2+^ were 25.12 mg·g^−1^ and 70.41 mg·g^−1^, respectively, representing 4.00 times and 2.75 times of WF. Magnesium modification elevated the sulfur content, pH, ash content, and electronegativity of MWF. The primary mechanisms behind MWF’s adsorption of Cd^2+^ and Pb^2+^ involve magnesium ion exchange and complexation with C=O/O=C–O, quaternary N, and S functional groups. MWF maintains robust stability and antioxidative properties, even with low aromaticity levels. Given the lower energy consumption during hydrochar production, MWF offers notable carbon sequestration benefits. The hydrothermal solution derived from MWF is nutrient-rich. Following supplementation with inorganic fertilizer, the hydrothermal solution of MWF significantly enhanced bok choy growth compared to the control group. In general, adopting magnesium-modified hydrothermal reactions to produce hydrochar and converting the resultant hydrothermal solution into water-soluble fertilizer proves a viable strategy for the eco-friendly utilization of chicken feathers. This approach carries substantial value for heavy metal remediation and agricultural practices.

## 1. Introduction

Heavy metal pollution for Cd and Pb is a global issue [1,2]. Cd and Pb in soil continuously accumulate through the food chain, causing adverse effects on human health [3]. Solidification and stabilization technology is commonly used to remediate Cd and Pb pollution in agricultural soils. This approach involves adding immobilizing agents to the soil to adsorb heavy metals or alter their forms, thereby reducing their bioavailability [4,5]. Biochar is a solid immobilizing material with a large specific surface area and abundant surface functional groups, widely used in the remediation of Cd, Pb, and other heavy metal-contaminated soils [6,7].

The adsorption performance of biochar is crucial for the remediation of soil Cd and Pb pollution. Most original biochars exhibit relatively weak adsorption performance, necessitating modifications to enhance their adsorption capacity [8]. Metal modification is a common modification method that can bind with heteroatoms such as S, N, and P in biomass, improving biochar’s adsorption performance [9]. And then, immobilizing agents used to remediate heavy metal-polluted soil only change the forms of heavy metals in the soil; the heavy metals remain in the soil, and the decomposition and transformation of immobilizing agents may lead to the re-release of heavy metals [10]. Therefore, the stability of immobilizing agents is crucial for long-term inhibition of heavy metals. The stability of biochar directly affects its persistence in soil, which is key to achieving long-term pollution control and preventing secondary pollution. It is also crucial for biochar to realize carbon sequestration benefits [11].

The preparation processes for biochar mainly include pyrolysis, gasification, and hydrothermal carbonization [12]. Among these, pyrolysis is the most commonly used method for biochar production, and its solid product better meets the typical definition of biochar. Hydrothermal carbonization (HTC) is carried out in the presence of water, making it unaffected by the high moisture content of raw materials, and its lower reaction temperature significantly reduces energy consumption [13,14]. HTC was employed as a low-energy consumption process for the production of carbon-rich heavy metal adsorbents.

Apart from a small amount of moisture, lipids, fibers, and ash, about 90% of feathers consist of keratin [15,16]. Keratin is an essential structural protein found in animal epithelial cells, primarily sourced from animal hair or hooves [17]. Most feathers are discarded or incinerated after being processed into feed or down products, resulting in a large amount of keratin waste [16]. Chickens’ feathers are a common type of avian feather, primarily composed of organic elements such as carbon (C), nitrogen (N), hydrogen (H), sulfur (S), and others (Table S1). Numerous sulfur-containing functional groups exhibit strong binding affinity with heavy metals such as cadmium and lead, with S being an essential constituent element in plant heavy metal detoxification [18,19,20,21]. Pyrolyzing chicken feathers into pyrolysis char has been proven to be a feasible measure [22,23]. But during the conventional high-temperature pyrolysis process for pyrolysis char production, S was significantly lost, whereas relatively low-temperature hydrothermal reactions for hydrochar could preserve S to a greater extent [18,24]. Currently, some studies have begun to explore the use of chicken feathers as a raw material for preparing hydrochar. Eliuz et al. (2022) prepared hydrochar using chicken feathers and loaded it with plant extracts as an antibacterial product [25]. Islam et al. (2021) synthesized hydrochar from chicken feathers for use as an adsorbent for phosphates [26]. Akter et al. (2021) have conducted preparation, characterization, and phytotoxicity evaluations of chicken feather-derived hydrochar [27]. Overall, existing studies predominantly employed pure water as a reaction solvent for preparing chicken feather-derived hydrochar, yet without effective modifications [25,26,27]. In additional, the utilization of chicken feather hydrothermal reaction liquids remains underexplored. A substantial number of nutrients were introduced into the hydrothermal reaction liquid during the process, rendering it nutrient-rich [28,29].

In general, unmodified hydrochars exhibit poor heavy metal adsorption capacity [30]. It is necessary to carry out modifications when preparing hydrochar for the purpose of heavy metal adsorption. The deposition of metal oxides and metal salts represents a novel method for altering the surface chemical properties of hydrochar and enhancing its adsorption capacity [31]. Metal elements such as zinc, iron, and magnesium (Mg) can bind with S, N [9]. Many studies have found that metal heteroatomic coordination bonds can improve material thermal resistance [32,33]. The improvement in thermal resistance may play a role in protecting heteroatoms. And then, Mg is a crucial medium element for plants. Synthesizing hydrothermal products through Mg-coupled hydrothermal reactions not only increased the adsorption capacity and S content of hydrochar but also enhanced the plant growth-promoting properties of the resultant hydrothermal products.

In this study, chicken feathers were used as the raw material. Mg served as a solvent for hydrothermal reactions to produce magnesium-modified high-sulfur hydrochar and hydrothermal reaction liquids containing Mg. The potential application value of hydrochar in soil environments was assessed through an analysis of its adsorption capacity (Cd^2+^ and Pb^2+^) and environmental stability. Additionally, the feasibility of utilizing hydrothermal reaction liquids as organic water-soluble fertilizers was evaluated through pot experiments using bok choy (*Brassica chinensis* L.).

## 2. Materials and Methods

### 2.1. Chemical Agent

The reagents used in this study, including hydrogen peroxide (H_2_O_2_), nitric acid (HNO_3_), and sodium nitrate (NaNO_3_), were purchased from the Guangzhou Chemical Reagent Factory. Magnesium chloride hexahydrate (MgCl_2_·6H_2_O), cadmium nitrate tetrahydrate (Cd(NO_3_)_2_·4H_2_O), and lead nitrate (Pb(NO_3_)_2_) were purchased from Macklin Reagent Company.

### 2.2. Preparation of High-Sulfur Hydrochar

The chicken feathers were sourced from Wen’s Group Co., Ltd. They were crushed into fluff using a grinder and then ground in a ball mill at 35 s^−1^ for 90 min and used as raw material for the hydrothermal reaction.

A 1 mol·L^−1^ Mg^2+^ solution was prepared using MgCl_2_·6H_2_O. A weight of 2.0 g of powdered chicken feathers was weighed and placed into a reaction vessel, and then 20 mL of ultrapure water or the 1 mol·L^−1^ Mg^2+^ solution was added. The mixture was stirred at 180 rpm in a shaker at 25 °C for 4 h. After sufficient stirring, the reaction vessel containing the inner liner was placed in an electrically heated convection oven with an average heating rate of 3 °C·min^−1^, heated to 180 °C, and maintained at that temperature for 240 min. After cooling to room temperature, the vessel was removed. The product in the inner liner was transferred to a 50 mL centrifuge tube and centrifuged at 8000 rpm for 10 min to separate the solid–liquid products. The liquid product was stored in a refrigerator for later use, while the solid product was washed three times with ultrapure water, dried in a freeze dryer, and sieved through a 60-mesh sieve. The hydrochar synthesized using magnesium as the hydrothermal reaction solution was named MWF, while those synthesized using ultrapure water as the hydrothermal reaction solution were named WF. The yield was calculated by the following equations:

(1) $$Yield(\%)=\frac{Weight\;of\;hydrochar\;}{Weight\;of\;biomass}\times 100\%$$

### 2.3. Characterization of Hydrochar

The pH of the materials was determined by immersion extraction at a solid-to-liquid ratio of 1:20. The cation exchange capacity (CEC) of hydrochar was measured using an improved ammonium acetate exchange method [34,35]. Ash content was determined by heating at 800 °C for 6 h in a muffle furnace, and the elements C, H, N, and S were analyzed using an elemental analyzer (Vario Micro Cube, Elementar, Hanau, Germany). The Fourier-transform infrared (FTIR) spectra of the materials were scanned in the wavelength range of 4000 cm^−1^ to 400 cm^−1^ using an FTIR analyzer (Tensor27, Bruker, Ettlingen, Germany). The porosity of hydrochar was determined by nitrogen adsorption using a surface area analyzer (ASAP-2460, Micromeritics, Norcross, USA). The nuclear magnetic resonance (NMR) procedure was as follows: ^13^C frequency of 100.63 MHz, magic angle spinning rate of 6.0 kHz, recycle time of 1.2 s, contact time of 1.2 ms. Measurements were conducted on a nuclear magnetic resonance spectrometer (Avance III 400MHZ, Bruker, Rheinstetten, Germany). Ultrapure water was used as the dispersant, and the Zeta potential of hydrochar at different pH levels (2–6) was measured using a Zeta analyzer (Zetasizer Nano ZS90, Malvern, Malvern, UK). Surface morphology was analyzed using a scanning electron microscope (SEM) (Evo Ma 15, Zeiss, Oberkochen, Germany) and energy-dispersive spectrometer (EDS) (Octane Plus, Edax, Mahwah, NJ, USA). Crystal structure analysis was performed using X-ray diffraction (XRD) (Ultima IV, Rigaku, Tokyo, Japan). Surface chemical composition was analyzed using X-ray photoelectron spectroscopy (XPS) (Escalab 250Xi, Thermo Fisher, Waltham, MA, USA).

### 2.4. Cd^2+^ and Pb^2+^ Adsorption Isotherm Experiment

In the adsorption experiments [22], the background solution was 0.01 mol·L^−1^ NaNO_3_. Cd^2+^ and Pb^2+^ solutions were prepared using Cd(NO_3_)_2_·4H_2_O and Pb(NO_3_)_2_, respectively, and the pH of the solutions was adjusted to 5.00 ± 0.01 using NaOH and HNO_3_. Each adsorption experiment was performed in triplicate, with 20 mg of adsorbent added to 20 mL of Cd^2+^ and Pb^2+^ solutions. The initial concentrations of Cd^2+^ were 1 mg·L^−1^, 5 mg·L^−1^, 10 mg·L^−1^, 25 mg·L^−1^, 40 mg·L^−1^, 55 mg·L^−1^, 75 mg·L^−1^, and the initial concentrations of Pb^2+^ were 1 mg·L^−1^, 5 mg·L^−1^, 25 mg·L^−1^, 50 mg·L^−1^, 75 mg·L^−1^, 100 mg·L^−1^, 150 mg·L^−1^. The mixture was shaken in a shaker at 25 °C and 180 rpm for 24 h. Afterward, the solution was filtered using a 0.45 μm filter. Depending on the specific situation, the filtrate was diluted with a 2% HNO_3_ solution, and the concentrations of Cd^2+^ and Pb^2+^ in the filtrate or dilute solution were determined using a flame atomic absorption spectrophotometer (AA-7000, Shimadzu, Kyoto, Japan). The concentration of Cd^2+^ and Pb^2+^ adsorbed on the hydrochar was calculated according to Equation (2):

(2) $$q_{e}=\frac{\left(C_{0}-C_{e}\right)V}{m}$$

where q_e_ (mg·g^−1^) is the amount of adsorbed Cd^2+^ and Pb^2+^ at equilibrium, V is the volume (L) of Cd^2+^ and Pb^2+^ solution, and m (g) is the weight of the hydrochar; C_0_ and C_e_ (mg·L^−1^) are the initial and equilibrium concentrations of Cd^2+^ and Pb^2+^ in the solution.

The isotherm model was fitted using OriginPro 2023 b. The isotherms were fitted and calculated using the Langmuir isotherm model Equation (3) and the Freundlich isotherm model Equation (4):

(3) $$q_{e}=q_{m}\frac{K_{L}C_{e}}{1+K_{L}C_{e}}$$

(4) $$q_{e}=K_{F}C_{e}^{\frac{1}{n}}$$

where q_e_ (mg·g^−1^) and q_m_ (mg·g^−1^) are the equilibrium and maximum adsorption amounts, C_e_ (mg·L^−1^) are the equilibrium concentrations of Cd^2+^ and Pb^2+^ in the solution, K_L_ (L·mg^−1^) is the Langmuir adsorption constant, K_F_ (mg^1−(1/n)^·L^1/n^·g^−1^) is the Freundlich adsorption constant, and 1/n is the adsorption favorability.

### 2.5. H_2_O_2_ Chemical Oxidation Experiment

The hydrochar chemical oxidation followed the method outlined by Cross and Sohi (2013) [36]. Hydrochar powder was pulverized using a ball mill (25 s^−1^) for 3 min. Approximately 0.1 g of carbon-containing hydrochar was weighed into a glass test tube. The test tube was dried and weighed, and then 7 mL of a 2% H_2_O_2_ solution was added. After thorough mixing, the test tube was placed in an 80 °C water bath, shaken three times daily, and heated until the moisture evaporated. The test tube was then removed, dried, and weighed to determine the element content. The hydrochar stability (B_S_) was calculated by the following equations:

(5) $$B_{S}=\frac{m_{a}\times C_{a}}{m_{b}\times C_{b}}\times 100\%$$

where m_a_ is the residual mass of hydrochar after oxidation, C_a_ is the carbon content of hydrochar after oxidation, m_b_ is the residual mass of hydrochar before oxidation, and C_b_ is the carbon content of hydrochar before oxidation.

### 2.6. Preparation of Chicken Feather Organic Water-Soluble Fertilizer

The liquid products separated during the preparation of hydrochar WF and MWF were, respectively, named WF organic water-soluble fertilizer and MWF organic water-soluble fertilizer. The determination of the organic matter content, Mg content, pH (1:200 dilution), and total nitrogen (TN), total phosphorus (TP) and total potassium (TK) of organic water-soluble fertilizer were all referenced to the Chinese Ministry of Agriculture’s organic water-soluble fertilizers [37].

Based on the TN, TP, and TK, the contents of the major nutrient elements N, P_2_O_5_, and K_2_O in organic water-soluble fertilizers WF and MWF were supplemented to 82.48 g·L^−1^, 31.05 g·L^−1^, and 53.34 g·L^−1^, respectively. This adjustment was achieved by incorporating urea (CH_4_N_2_O), potassium dihydrogen phosphate (KH₂PO₄), and potassium chloride (KCl) as the three inorganic fertilizers. Additionally, the pH was measured (1:200 dilution), leading to the designation of WFNPK organic water-soluble fertilizer and MWFNPK organic water-soluble fertilizer, respectively.

### 2.7. Pot Experiment with Bok Choy

The soil samples were collected from paddy fields in Qujiang District, Shaoguan City, Guangdong Province. The soil samples were naturally air-dried, purified to remove impurities, sieved through a 5-mesh sieve, thoroughly mixed, and then used for pot experiments with bok choy. The nutrient content and basic physicochemical properties of the soil are shown in Table S2.

The potted experiments of bok choy were divided into five treatments: CK, NPK, WF, WFNPK, and MWFNPK, with three replicates for each treatment. The treatments were as follows: (1) CK served as the control group, receiving only water in the same amount as other treatments. (2) NPK involved the application of inorganic fertilizers, including CH_4_N_2_O, KH_2_PO_4_, and KCl, at a nutrient content of 137.47 mg N, 51.75 mg P_2_O_5_, and 88.91 mg K_2_O per pot. The fertilizers were evenly mixed and applied, with 40% as base fertilizer, 15% as first topdressing, 30% as second topdressing, and 15% as third topdressing, dissolved in water before application and applied every 7 days (Table S3). (3) WF utilized organic water-soluble fertilizers. (4) WFNPK involved the application of organic water-soluble fertilizers combined with NPK. (5) MWFNPK applied organic water-soluble fertilizers combined with NPK. All organic water-soluble fertilizers were diluted 200 times before application, with each pot receiving a total of 333.33 mL of diluted organic water-soluble fertilizer, following the fertilization method of NPK treatment, as detailed in Table S3.

Each replicate contained 2.0 kg of soil and three bok choy plants, supplemented with plant growth lights every evening and watered daily, lasting for 28 d.

### 2.8. Determination of Yield-Related Indicators for Bok Choy

The determination of bok choy primarily focused on yield-related indicators, including (1) the number of leaves of bok choy (excluding cotyledons); (2) plant height, maximum leaf length, corresponding leaf width, and root length; and (3) calculating the above-ground fresh weight and dry weight of bok choy through weighing methods.

## 3. Results and Discussion

### 3.1. Basic Characterization and Morphology Analysis of Hydrochar

The basic physicochemical properties of WF and MWF are shown in Table 1. The yields of chicken feather hydrochar WF and MWF were 15.90% and 16.76%, respectively. The addition of Mg slightly increased the yield of hydrochar, which was also reflected in the higher ash content of hydrochar MWF (17.42%). The ash content of hydrochar mainly comes from basic ions [38], while feathers are primarily composed of keratin, containing only a small amount of ash, fiber, and fat [15,16]. Therefore, it can be considered that the introduction of Mg metal increased the ash content of MWF and thus improved the yield. Some experimental results indicate that increasing basic ions and the ash content can raise the pH of hydrochar [39]. This may also be the reason for the higher pH of MWF (7.39) in this study, while the pH of hydrochar WF was acidic at 6.38.

The S content of raw hydrochar WF and MWF reached 3.34% and 3.68%, respectively. Chen et al. (2019) utilized chicken feathers to synthesize pyrolysis char, with a S content of only 0.32% [22]. Islam et al. (2021) employed chicken feathers to synthesize hydrochar, resulting in a S content of 1.88% [26]. Furthermore, Li et al. (2022) employed sludge as the raw material for hydrochar preparation; its S content was also low, at only 0.31% [40]. WF and MWF were significantly higher than the S content of chicken feather pyrolysis char and other hydrochar in many studies (Table S4). This is related to the characteristics of the hydrothermal reaction. Compared to pyrolysis char, hydrochar operates at lower temperatures and in a more closed reaction system. When the hydrothermal reaction temperature is low (180–300 °C), S primarily converts between the solid and liquid phases of the reaction system; thus, hydrochar can retain more S [24]. In contrast, the S content in pyrolysis char is usually lower, generally not exceeding 1.4% [18]. In this study, the addition of Mg further increased the content of heteroatoms N and S in hydrochar, with elemental contents of N and S in hydrochar MWF reaching 7.33% and 3.68%, respectively, representing increases of 32.05% and 10.10% compared to WF. This may be because Mg bonds with heteroatoms N and S, thereby reducing the loss of N and S in solid products. The enriched N and S can enhance the adsorption capacity and nutrient value of hydrochar. Additionally, S is also an important element for detoxifying heavy metals in crops [41]. Therefore, MWF has a higher environmental and agricultural application value. The carbon contents of hydrochar WF and MWF were 51.08% and 52.03%, respectively, with H/C molar ratios of 1.71 and 1.73. It can be observed that there is no significant difference in these two indicators between the two hydrochars. The H/C molar ratio is an important indicator reflecting the aromaticity and stability of hydrochar [42]; the carbon content and stability of hydrochar collectively influence its carbon sequestration effect [43]. Considering the higher yield of MWF, it can be inferred that the chelation of Mg not only increased the content of heteroatoms (N, S) but also did not decrease its carbon sequestration effect and may have slightly increased it.

**Figure 1:**
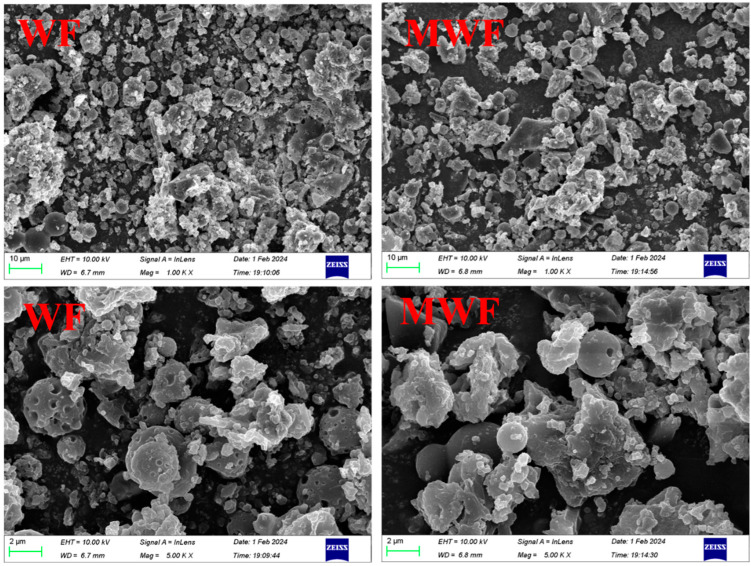
SEM images of unmodified hydrochar WF and magnesium-modified hydrochar MWF.

In terms of the microstructure, the specific surface area, pore volume, and pore size of hydrochar MWF were 1.267 m^2^·g^−1,^ 0.004 cm^3^·g^−1^, and 13.269 nm, respectively, while those of hydrochar WF were 2.049 m^2^·g^−1^, 0.002 cm^3^·g^−1^, and 5.718 nm, respectively. MWF has a smaller specific surface area but larger pore volume and pore size, indicating that the addition of MgCl_2_ may promote the development of large pores in hydrochar, thereby affecting its specific surface area. Figure 1 shows the SEM images of hydrochar, revealing numerous small spheres on the surfaces of WF and MWF, likely formed during the hydrothermal reaction. Compared to MWF, WF surfaces were denser, with more uneven surfaces and more pores on the carbon spheres, while MWF surfaces were relatively smooth, possibly due to the filling of pores by Mg elements. These phenomena indirectly indicate that WF may have a higher specific surface area. Additionally, SEM-EDS results (Figure S1) demonstrate that Mg was successfully loaded onto high-sulfur hydrochar MWF. The Mg-point position of MWF had a good coincidence degree with the N-point position and a certain coincidence degree with the S-point position. This was consistent with the increase in the N (32.05%) and S (10.10%) contents of MWF, suggesting a positive effect of magnesium on the N and S contents of MWF.

### 3.2. Adsorption Isotherms of Hydrochar

**Figure 2:**
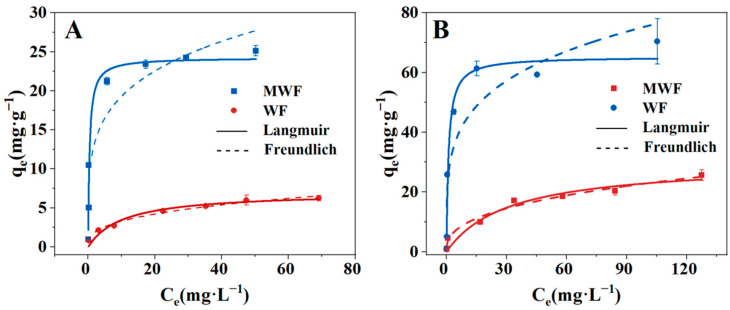
Fitting results of the adsorption isotherm for Cd^2+^ (**A**) and Pb^2+^ (**B**) adsorbed by WF and MWF.

The adsorption isotherms of WF and MWF are presented in Figure 2, and the specific parameters of the Langmuir and Freundlich models are detailed in Table 2. The Langmuir model is an empirical model describing monolayer adsorption, suggesting that the adsorption behavior of materials is chemical in nature [44,45]. The Freundlich model describes multilayer adsorption, considering the adsorption behavior of materials as physical adsorption [45,46]. The adsorption isotherms of Cd^2+^ and Pb^2+^ by hydrochar WF better fit the Freundlich model (R^2^ of 0.986 and 0.976), indicating that the main adsorption mechanism of WF for Cd^2+^ and Pb^2+^ is physical adsorption. Magnesium-modified high-sulfur hydrochar MWF shows a better fit to the Langmuir model for adsorbing Cd^2+^ and Pb^2+^, indicating that the main adsorption mechanism of MWF for Cd^2+^ and Pb^2+^ is chemical adsorption. The maximum equilibrium adsorption amounts (q_em_) of MWF for Cd^2+^ and Pb^2+^ were 25.12 mg·g^−1^ and 70.41 mg·g^−1^, respectively. The q_em_ of WF for Cd^2+^ and Pb^2+^ was 6.25 mg·g^−1^ and 25.65 mg·g^−1^, respectively. MWF showing increases of 300.40% and 174.50% in q_em_ for Cd^2+^ and Pb^2+^, respectively, compared to WF. The results shows that Mg modification could improve the adsorption capacity of Cd^2+^ and Pb^2+^ in chicken feather hydrochar.

MWF q_em_ for Cd^2+^ and Pb^2+^ was compared with other chicken feather adsorbents reported in the literature, including raw feather, chemically activated feather, and feather-based pyrolysis char [22,23,47,48,49,50]. Additionally, MWF q_em_ for Cd^2+^ and Pb^2+^ was compared with other non-chicken-feather pyrolysis char and hydrochar [51,52,53]. MWF is in a mid-range position, indicating the feasibility of hydrochar MWF as a heavy metal adsorbent.

### 3.3. Possible Adsorption Mechanisms of Hydrochar

#### 3.3.1. SEM-EDS

The SEM-EDS images of WF and MWF after adsorption of Cd^2+^ and Pb^2+^ are shown in Figure S2. After the adsorption of Cd^2+^ and Pb^2+^ by WF and MWF, the surface successfully detected Cd^2+^ and Pb^2+^ elements, indicating that both WF and MWF have a certain adsorption capacity for Cd^2+^ and Pb^2+^. Additionally, the Cd^2+^ and Pb^2+^ elements on the surface of MWF were significantly higher than those on WF, which demonstrates the stronger Cd^2+^ and Pb^2+^ adsorption capacity of MWF.

#### 3.3.2. Zeta Potential

**Figure 3:**
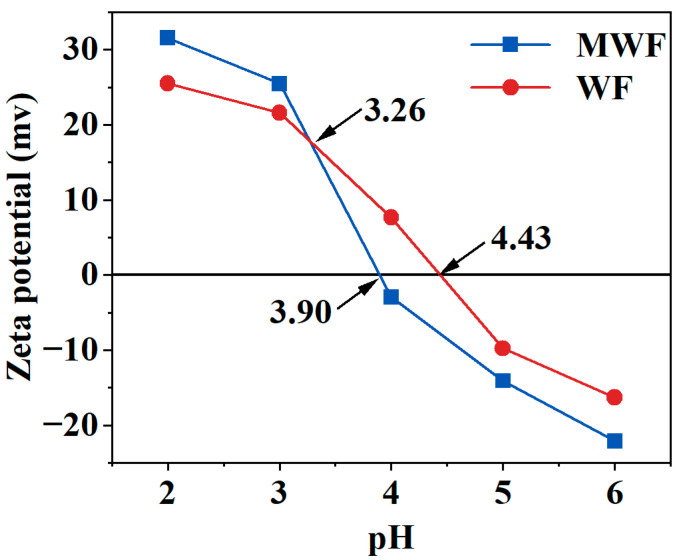
Zeta potentials of hydrochar WF and MWF under different pH (2–6) pure water.

The surface charge characteristics of materials can affect the adsorption performance of hydrochar. When adsorbing heavy metal cations Cd^2+^ and Pb^2+^, a higher amount of negative surface charge on the material is more favorable for adsorbing these two cations [54]. The Zeta potentials of WF and MWF at different pH values (2–6) are shown in Figure 3. At pH below 3.26, WF has a lower Zeta potential, but as the pH increases, the number of negative surface charges on MWF exceeds that of WF. The isoelectric points of MWF and WF were 3.90 and 4.43, respectively, with MWF having a lower isoelectric point. The initial pH of the solution in this study was 5.00 ± 0.01; therefore, under the adsorption conditions of this study, MWF has more negative surface charges compared to WF. Consequently, MWF adsorbs more of the heavy metal ions Cd^2+^ and Pb^2+^ through electrostatic adsorption compared to WF. However, based on the adsorption isotherm fitting results, it is believed that the main adsorption mechanism of MWF is chemical adsorption, while that of WF is primarily physical adsorption. Therefore, the electrostatic adsorption of the heavy metal ions Cd^2+^ and Pb^2+^ by MWF should be considered an important aspect of MWF’s adsorption of Cd^2+^ and Pb^2+^, but not the primary adsorption mechanism. Hence, further analysis of MWF’s adsorption mechanism was conducted using FTIR, XRD, and XPS.

#### 3.3.3. FTIR Analysis

The FTIR spectra of WF and MWF before and after adsorption of Cd^2+^ and Pb^2+^ are shown in Figure S3. The results indicate that both WF and MWF formed broad peaks of overlapping O-H and N-H stretching vibrations near 3300 cm^−1^, with O-H and N-H peaks at 3283 cm^−1^ for WF and shifted to 3287 cm^−1^ for MWF, possibly due to Mg modification. The characteristic peak representing C=O shifted from 1636 cm^−1^ for WF to 1638 cm^−1^ for MWF. The peaks corresponding to amide II groups and N-H bending also shifted, from 1543 cm^−1^ for WF to 1547 cm^−1^ for MWF. The C-O bond characteristic peak for WF appeared at 1037 cm^−1^, while for MWF, it appeared at 1098 cm^−1^, indicating a significant influence of Mg addition on the formation of C-O bonds. Finally, WF and MWF exhibited strong double peaks between 600 and 650 cm^−1^, possibly due to C=S bond stretching vibrations [55]. Furthermore, several weak characteristic peaks appeared for MWF in the range of 700–800 cm^−1^ (WF did not), which may be attributed to the coordination of Mg with S-O bonds. However, after adsorption, only one characteristic peak was observed at ~700 cm^−1^, possibly resulting from the destruction of the coordination bond between Mg and S after adsorption. These findings provide indirect evidence that Mg can bond with sulfur functional groups, thereby protecting sulfur elements.

Elsewhere, after adsorption of Cd^2+^ and Pb^2+^, there were slight changes and variations in intensity in the characteristic peaks of O-H bonds for both WF and MWF. The characteristic peak of C=O shifted after adsorption of Cd^2+^ and Pb^2+^, with WF peaks shifting from 1636 cm^−1^ to 1646 cm^−1^ and 1628 cm^−1^, and MWF peaks shifting from 1638 cm^−1^ to 1640 cm^−1^ and 1636 cm^−1^, with a noticeable decrease in intensity for WF after Cd^2+^ adsorption. The N-H bending peak shifted from 1543 cm^−1^ for WF to 1550 cm^−1^ after adsorption, while for MWF, it shifted from 1547 cm^−1^ to 1539 cm^−1^ and 1540 cm^−1^. The characteristic peak of C-O bonds showed minimal change after Cd^2+^ adsorption for WF, but disappeared directly after Pb^2+^ adsorption, while for MWF after Cd^2+^ and Pb^2+^ adsorption, slight changes were observed along with the emergence of new peaks nearby. The C=S bond stretching significant shifts after Cd^2+^ adsorption for WF, while the change was smaller after Pb^2+^ adsorption; for MWF, slight shifts were observed after Cd^2+^ and Pb^2+^ adsorption. In summary, the O-H, N-H, C-O, and C=S functional groups of hydrochar MWF underwent complexation reactions with Cd^2+^ or Pb^2+^, thereby adsorbing heavy metals. These chemical bonds are considered adsorption sites for the heavy metals Cd^2+^ and Pb^2+^ in MWF.

#### 3.3.4. XRD

The XRD patterns of WF and MWF before and after adsorption of Cd^2+^ and Pb^2+^ are shown in Figure S4. There were no significant differences in the XRD spectra of the two hydrochars, and no new peaks were observed in the XRD spectra after Cd^2+^ and Pb^2+^ adsorption on WF and MWF. Therefore, precipitation is not the main adsorption mechanism of WF and MWF. FTIR analysis revealed functional group complexation reactions, and SEM-EDS results indicated the successful loading of Mg elements onto MWF. Therefore, XPS analysis was further conducted to investigate whether Mg ion exchange and functional group chelation occurred in WF and MWF.

#### 3.3.5. XPS

**Figure 4:**
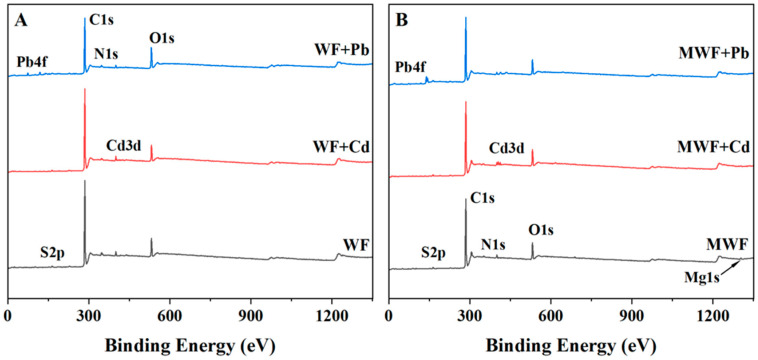
XPS spectra of hydrochar WF (**A**) and MWF (**B**) before and after adsorption of Cd^2+^ and Pb^2+^.

The elemental atomic ratios of Cd^2+^ and Pb^2+^ adsorption on WF and MWF are shown in Table 3. After adsorbing Cd^2+^ and Pb^2+^, the atomic ratios of Cd^2+^ and Pb^2+^ on WF were 0.10 and 0.09, respectively, while on MWF, they were 0.31 and 0.44, respectively. It can be observed that after adsorption, MWF had higher atomic ratios of Cd^2+^ and Pb^2+^, indicating that MWF adsorbed more Cd^2+^ and Pb^2+^ and had a stronger adsorption capacity. The atomic ratio of Mg on MWF before adsorption was 0.48%, while WF surfaces did not contain Mg atoms, consistent with SEM-EDS results, demonstrating successful loading of Mg atoms onto MWF. However, after adsorbing Cd^2+^ and Pb^2+^, the atomic ratios of Mg decreased to 0.31% and 0.23%, respectively, on MWF. This decrease may be attributed to ion exchange interactions between Mg and Cd/Pb during adsorption, which is further supported by the XPS spectrum in Figure 4 showing a reduction in Mg signal values after adsorption on MWF, reinforcing this observation.

The O1s, N1s, S2p, C1s, and Mg1s spectra before and after the adsorption of Cd^2+^ and Pb^2+^ for WF and MWF are shown in Figures S5 and S6. After adsorption, the O1s, N1s, and S2p characteristic peaks all changed for WF and MWF (N1s remained unchanged after Pb^2+^ adsorption for WF). This indicates that functional groups containing N, O, and S elements participated in the adsorption process of both WF and MWF, which is consistent with the FTIR results. Additionally, the peak changes in MWF after adsorption were significantly greater than those in WF, which may be attributed to the greater chelation of Cd^2+^ and Pb^2+^ by the aforementioned functional groups in MWF, resulting in more pronounced changes. Furthermore, no significant Mg peak was observed in WF before or after adsorption. In contrast, after adsorption of Cd^2+^ and Pb^2+^, the Mg peak of MWF noticeably decreased, indicating further evidence of an exchange interaction between magnesium ions and Cd^2+^/Pb^2+^.

The C1s XPS spectra of hydrochar WF and MWF before and after adsorption are shown in Figure S7A,B. Before adsorption, the C1 peak of WF and MWF was divided into five characteristic peaks: sp^2^ C=C (283.78–283.83 eV), sp^3^ C-C (284.58–284.68 eV), C-O/C-N (285.13–285.31 eV), C=O (285.83–285.97 eV), and O=C-O (287.98–288.04 eV) [56,57]. After adsorption, sp^2^ C=C, sp3 C–C, and C–O/C–N showed minor changes, while C=O and O=C–O exhibited significant changes. This indicates that C=O/O=C–O are important sites for Cd^2+^ and Pb^2+^ adsorption on hydrochar WF and MWF.

The N1s XPS spectra of hydrochar WF and MWF before and after adsorption are shown in Figure S7C,D. Before adsorption, the C1 peak of WF and MWF was divided into four characteristic peaks: pyridinic N (398.78–398.93 eV), pyridonic N (399.63–399.67 eV), pyrrolic N (400.33–400.43 eV), and quaternary N (401.13–401.83 eV) [58,59]. After adsorption, pyridinic N, pyridonic N, and pyrrolic N showed minor changes, while quaternary N underwent significant changes. This indicates that quaternary N is an important site for Cd^2+^ and Pb^2+^ adsorption on hydrochar WF and MWF.

**Figure 5:**
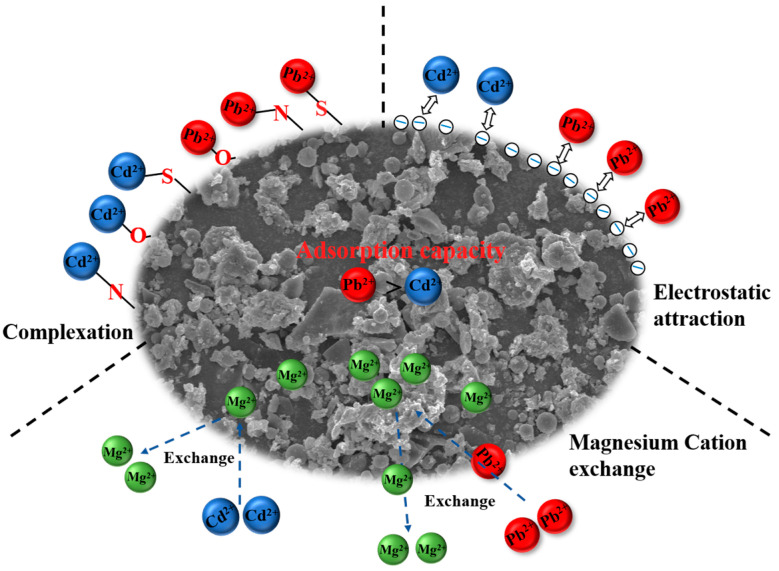
Mechanism diagram of adsorption of Cd^2+^ and Pb^2+^ by hydrochar MWF.

In summary, during the adsorption of Cd^2+^ and Pb^2+^ by MWF, ion exchange involving Mg occurred, and C=O/O=C–O, quaternary N, and S functional groups underwent chelation reactions with Cd^2+^ and Pb^2+^, serving as important adsorption sites for MWF. Finally, the adsorption mechanisms of Cd^2+^ and Pb^2+^ by MWF are shown in Figure 5.

### 3.4. Environmental Stability of Hydrochar

#### 3.4.1. NMR

**Figure 6:**
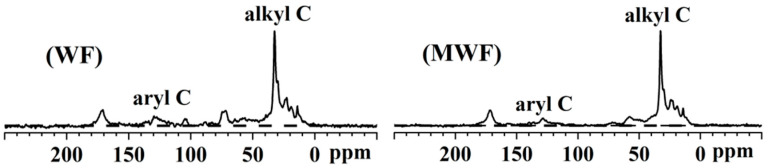
^13^C CP/TOSS spectra of hydrochar WF and MWF.

According to previous studies, the spectrum was integrated into resonance regions of alkyl C (0–45 ppm), O/N-alkyl C (45–110 ppm), aromatic C (110–165 ppm), and carboxyl/carbonyl/amide C (165–220 ppm) [60,61,62,63]. The ^13^C CP/TOSS NMR spectra of WF and MWF are shown in Figure 6. All spectra of hydrochars exhibited a dominant alkyl peak at 30 ppm, while the aromatic peak range representing aromatic compounds (110–160 ppm) was significantly smaller than the aliphatic peak. This is contrary to pyrolysis char, where the aromatic peak dominated completely [60,61,62,63,64,65]. However, this consistency with the higher H/C and O/C molar ratios of hydrochar WF and MWF was due to the lower hydrothermal reaction temperature in this study (180 °C). In the alkyl region (0–45 ppm), a strong signal near 30 ppm was attributed to methylene (CH_2_), while a weaker signal near 20 ppm was attributed to methyl (CH_3_). Additionally, strong signal peaks attributed to COOR/CH_3_COO groups were observed in the range of 160–180 ppm for hydrochar WF and MWF.

The functional group compositions of hydrochar WF and MWF are shown in Table 4. The proportions of alkyl C, O/N-alkyl C, aromatic C, and carboxyl/carbonyl/amide C functional groups in hydrochar WF were 57.90%, 22.05%, 2.15%, and 7.90%, respectively. For MWF, the proportions of alkyl C, O/N-alkyl C, aromatic C, and carboxyl/carbonyl/amide C functional groups were 63.88%, 14.11%, 11.64%, and 10.38%, respectively. Aromaticity is the ratio of aromatic carbon to all carbon elements, which is an important indicator for evaluating the stability of hydrochar and can be approximated by the H/C molar ratio [42]. The aromatic C content of WF was slightly higher than that of MWF, consistent with W’s slightly lower H/C molar ratio, indicating that WF may have slightly higher environmental stability than MWF. Alkyl group carbon is the part of hydrochar resulting from incomplete pyrolysis, with the alkyl C content of MWF higher than WF and the O/N-alkyl C content lower than WF. This was consistent with MW’s’ lower O and N elements, where the sum of O and N elements in MWF was 19.36% compared to 23.59% in WF. Additionally, the carboxyl/carbonyl/amide C content was higher in MWF than in WF. Carboxyl/carbonyl/amide C is a hydrophilic functional group, while alkyl C, O/N-alkyl C, and aromatic C are hydrophobic functional groups. An increase in hydrophobic groups can enhance the antioxidant properties of hydrochar [66]. The proportions of hydrophobic groups in carbon elements for WF and MWF were 92.10% and 89.62%, respectively, with the hydrophobic group content higher in hydrochar WF than in MWF. Molecular hydrophobicity originates from the non-polarity of the molecule itself, with the non-polarity of O/N-alkyl C weaker than that of alkyl C. WF had an O/N-alkyl C content of 22.05%, while MW’s’ O/N-alkyl C proportion was 14.11%. Considering W’s’ higher proportion of hydrophobic groups in carbon elements and other unknown hydrophobic group content, the impact of hydrophobicity on the antioxidant properties of WF and MWF is still inconclusive. In summary, the aromatic C contents of the two hydrochars were relatively close, suggesting similar stability between WF and MWF. However, considering the differences in hydrophobic group proportions and composition, further validation of the antioxidant properties of WF and MWF is needed through chemical oxidation experiments.

#### 3.4.2. H_2_O_2_ Chemical Oxidation Experiment

Table S5 presents the results of the hydrochar H_2_O_2_ stability assessment. The carbon loss rates of hydrochar WF and MWF were 41.84% and 40.58%, respectively, indicating carbon stability of 58.16% and 59.42%. The antioxidant stability of hydrochar MWF and WF was generally similar, with MW’s carbon loss rate slightly increasing by 1.26% compared to WF, but without significant differences (*p* < 0.05). This is largely consistent with the similar aromaticity and H/C molar ratios of the two hydrochars, reflecting the combined influence of hydrochar aromaticity and hydrophobicity. The aromaticity of hydrochar WF and MWF was only 12.15% and 11.64%, respectively. Compared with pyrolysis char, hydrochar WF and MWF still have stronger resistance to H_2_O_2_ oxidation with relatively low aromaticity [36]. This is related to the numerous hydrophobic groups on the surface of the hydrochar, which enhance its antioxidant properties [66]. Compared to pyrolysis char, which is a product of high-temperature pyrolysis, hydrochar production occurs at lower temperatures, significantly saving energy consumption while maintaining good antioxidant properties. Therefore, both hydrochars WF and MWF in this study exhibit relatively high stability and carbon sequestration values.

### 3.5. Composition of Organic Water-Soluble Fertilizer

The properties and nutrient composition of organic water-soluble fertilizers are shown in Table S6. The organic matter content of organic water-soluble fertilizers WF and MWF was 49.68 g·L^−1^ and 53.84 g·L^−1^, respectively, indicating a relatively high organic matter content. The original organic water-soluble fertilizers WF and MWF had low TP and TK contents, while the TN content was relatively high, reaching 9.84 g·L^−1^ and 7.37 g·L^−1^, respectively, consistent with the high nitrogen content in chicken feathers (Table S1). The TN content in MWF organic water-soluble fertilizer was lower than WF, possibly due to the inhibitory effect of Mg^2+^ on N element complex gasification in chicken feathers. The higher nitrogen content in magnesium-modified hydrochar (MWF) (Table 1) also indirectly supports this viewpoint.

From the pH (1:200) perspective, the original organic water-soluble fertilizers had weak alkalinity, but after adding inorganic fertilizers, the pH of organic water-soluble fertilizers decreased slightly. The pH of WFNPK and MWFNPK were 6.360 and 5.627, respectively, attributed to the acidity of KH₂PO₄ when dissolved in water, leading to a decrease in the pH of organic water-soluble fertilizers. On the other hand, the pH of Mg-containing organic water-soluble fertilizers was slightly lower than that of ordinary organic water-soluble fertilizers, likely due to the influence of Mg^2+^. In an aqueous solution, Mg^2+^ undergoes hydrolysis reactions, producing a certain amount of H^+^, thereby reducing the pH of water-soluble fertilizers.

Overall, the original organic water-soluble fertilizers had a high organic matter and TN, making them valuable for use. However, they had low levels of major nutrient elements P and K, requiring supplementation of inorganic nutrient elements. The Mg hydrothermal reaction provided a large amount of Mg nutrient elements (22.69 g·L^−1^) to water-soluble fertilizers, further enhancing the value of organic water-soluble fertilizers. However, the specific fertilizer efficiency still needs further verification through potted plant experiments.

### 3.6. Effects of Organic Water-Soluble Fertilizer on the Growth of Bok Choy

The growth status of bok choy is shown in Figure S6. The growth of bok choy under MWFNPK and WFNPK treatments was robust, while the bok choy under CK treatment appeared slender and weak. Bok choy under the WF and NPK treatments showed better growth than under CK, with similar growth patterns between the two treatments.

**Figure 7:**
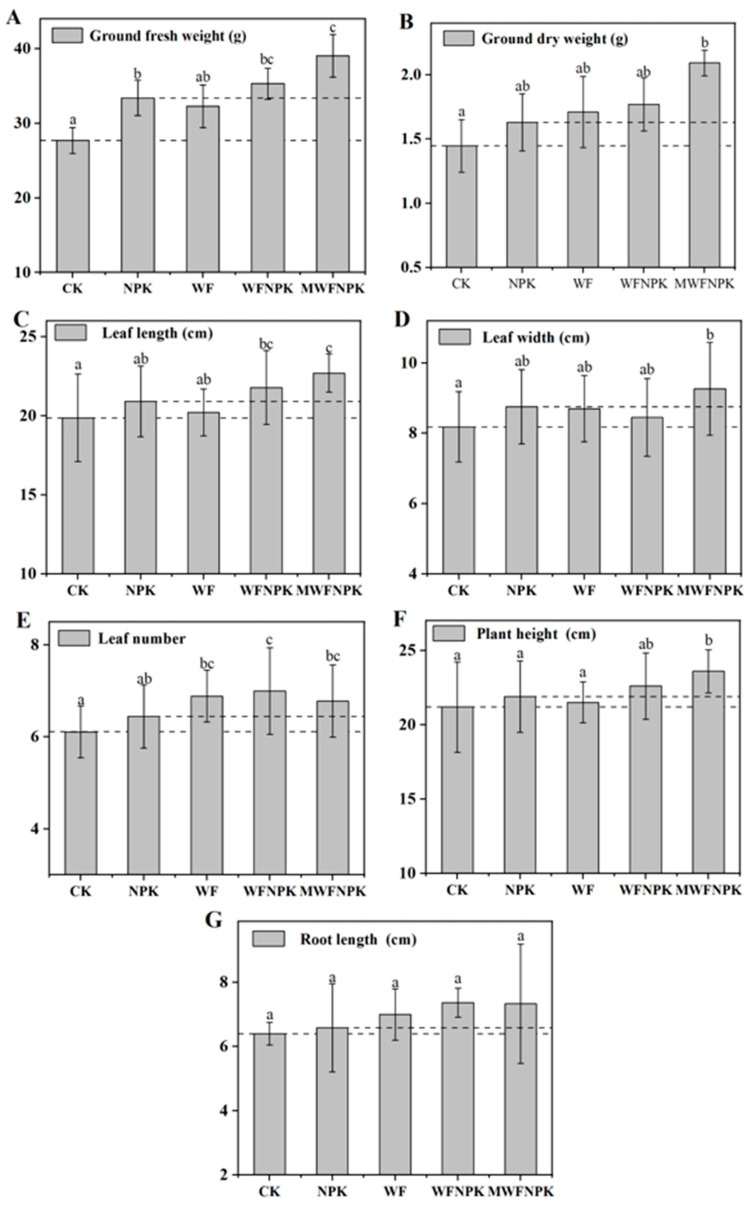
Effects of different fertilization methods on aboveground fresh weight (**A**), aboveground dry weight (**B**), leaf length (**C**), leaf width (**D**), leaf number (**E**), plant height (**F**) and root length (**G**) of bok choy. Different lowercase letters in the figure indicate significant differences between treatments (*p* < 0.05).

Under different fertilization treatments, yield-related indicators of bok choy are presented in Figure 7 and Table S7. All organic water-soluble fertilizer treatments exhibited certain growth-promoting effects. Among them, the growth-promoting effect of the WF treatment was similar to that of the NPK treatment. However, in terms of aboveground dry weight, leaf number, and root length, the WF treatment outperformed the NPK treatment, while in terms of aboveground fresh weight, leaf length, leaf width, and plant height, the WF treatment was slightly inferior to the NPK treatment. Generally, organic fertilizers have lower nutrient concentrations (Table S6) and slower effects, while inorganic fertilizers have quicker nutrient effects. In this study, the growth-promoting effect of the WF organic water-soluble fertilizer treatment was similar to the NPK treatment. This may partly be due to the high content of readily available nutrients in the potting soil (Table S2), but compared to the CK treatment, the WF treatment showed improvements in various yield indicators, indicating the feasibility of chicken feather hydrothermal liquid as an organic water-soluble fertilizer.

Organic–inorganic compound fertilizers are commonly used to increase crop yields and fertilizer utilization efficiency, providing balanced nutrition for crops. In this study, the WFNPK treatment (organic-inorganic compound fertilizer) further enhanced the growth-promoting effect compared to the WF treatment (organic fertilizer). It outperformed the CK and NPK treatments in all indicators except leaf width, further demonstrating the feasibility of chicken feather hydrothermal liquid as an organic water-soluble fertilizer. Moreover, the growth-promoting effect of the MWFNPK treatment was further enhanced compared to the WFNPK treatment. Compared to the CK treatment, the MWFNPK treatment significantly (*p* < 0.05) increased the aboveground fresh weight, leaf length, leaf width, leaf number, and plant height of bok choy by 40.90%, 44.62%, 14.20%, 13.24%, 10.91%, and 11.35%, respectively. Compared to the NPK treatment, the MWFNPK treatment significantly (*p* < 0.05) increased the aboveground fresh weight, leaf length, and plant height of bok choy by 16.91%, 14.20%, and 7.79%, respectively. The growth-promoting effect of the MWFNPK treatment not only surpassed the use of inorganic fertilizer (NPK treatment) but also exceeded other organic fertilizer treatments (WF and MWF treatments). This may be related to the high content of the metallic nutrient Mg in the MWFNPK water-soluble fertilizer (Table S6). The metallic nutrient Mg is not only an important component of plant chlorophyll but also promotes the metabolism and absorption of nutrients such as nitrogen and phosphorus in plants [67].

Finally, in terms of root length, the root lengths of bok choy under the CK and NPK treatments were similar at 6.40 cm and 6.58 cm, respectively. Under organic water-soluble fertilizer treatments, the root lengths of bok choy under the WF, WFNPK, and MWFNPK treatments were 7.00 cm, 7.37 cm, and 7.33 cm, respectively, representing increases of 9.37%, 15.10%, and 14.58% compared to the CK treatment. This phenomenon indicates that the chicken feather organic water-soluble fertilizer promoted the development of bok choy roots and may have subsequently promoted the growth and development of bok choy. In conclusion, chicken feather organic water-soluble fertilizer has high agricultural value, contains rich organic nutrients, and has demonstrated good growth-promoting effects on bok choy while reducing the use of inorganic fertilizers. The growth-promoting effect of organic–inorganic compound water-soluble fertilizers was further enhanced, with Mg-containing organic water-soluble fertilizer (MWFNPK) showing the best growth-promoting effect, effectively utilizing the residual liquid metallic nutrient Mg from magnesium-modified hydrothermal reactions.

## 4. Conclusions

High-sulfur hydrochar MWF was synthesized via a magnesium-modified coupling hydrothermal reaction, achieving a S content of 3.68%. The q_em_ values of MWF for Cd^2+^ and Pb^2+^ were 25.12 mg·g^−1^ and 70.41 mg·g^−1^, respectively, representing 4.00 times and 2.75 times those of WF. The main adsorption mechanisms of MWF included Mg ion exchange and complexation with C=O/O=C–O, quaternary N, and S functional groups. MWF exhibited lower aromaticity but demonstrated good antioxidant properties. Considering the lower production energy consumption of hydrochar, MWF showed promising carbon sequestration benefits. Thus, MWF may be used as a choice for remediating soil heavy metal pollution caused by Cd and Pb. After supplementation with inorganic fertilizers, magnesium-containing organic water-soluble fertilizer (MWFNPK) resulted in the optimal growth promotion of bok choy. Compared to the CK treatment, the MWFNPK treatment significantly (*p* < 0.05) increased the fresh weight, dry weight, leaf length, leaf width, leaf number, and plant height of bok choy by 40.90%, 44.62%, 14.20%, 13.24%, 10.91%, and 11.35%, respectively. In conclusion, preparing hydrochar through magnesium modification and converting hydrothermal solution into water-soluble fertilizer is a viable approach for the green utilization of chicken feathers. But MWF’s could not directly remove heavy metals such as cadmium and lead from the soil. Loading MWF onto recyclable gels is a feasible direction for future research.

## Figures and Tables

**Table 1:** Basic characterization of hydrochar WF and MWF.

Project	Index	Units	WF	MWF
Yield rate	/	%	15.90	16.76
pH	/	/	6.38	7.39
Elemental composition	C	wt.%	51.08	52.03
	H		7.28	7.51
	N		5.55	7.33
	S		3.34	3.68
	O ^a^		18.03	12.03
Ash	/	wt.%	14.72	17.42
Elemental molar ratio	H/C	/	1.71	1.73
	O/C	/	0.26	0.25
Microstructure properties	Surface area	m^2^·g^−1^	2.049	1.267
	Pore volume	cm^3^·g^−1^	0.002	0.004
	Pore size	nm	5.718	13.269

^a^ Calculate by difference subtraction.

**Table 2:** Langmuir and Freundlich isotherm fitting parameters for Cd^2+^ and Pb^2+^ adsorption by hydrochar WF and MWF.

Adsorbate	Material	Langmuir Model			Freundlich Model		
		q_m_ (mg·g^−1^)	K_L_ (L·g^−1^)	R^2^	K_F_ (mg^1-(1/n)^·L^1/n^·g^−1^)	1/n	R^2^
Cd	WF	7.1	0.096	0.950	1.465	0.353	0.986
	MWF	24.3	2.375	0.954	11.496	0.225	0.871
Pb	WF	30.1	0.032	0.939	4.006	0.379	0.976
	MWF	65.3	0.935	0.963	26.125	0.230	0.820

**Table 3:** The surface elemental atomic ratios (%) of WF and MWF before and after adsorption of Cd^2+^ and Pb^2+^ by XPS.

Elemental	WF	WF+Cd	WF+Pb	MWF	MWF+Cd	MWF+Pb
C	85.22	86.66	79.59	84.94	85.75	85.26
N	3.92	3.47	3.1	3.21	3.01	2.49
O	9.69	8.69	16.27	10.22	9.47	10.20
S	1.18	1.08	0.96	1.15	1.15	1.38
Mg	/	/	/	0.48	0.31	0.23
Cd	/	0.10	/	/	0.31	/
Pb	/	/	0.09	/	/	0.44

**Table 4:** The functional group composition (%) of hydrochar based on CP/TOSS analysis.

	Carboxyl/Carbonyl/Amide C	Aryl C	O/N-Alkyl C	Alkyl C
Hydrochar	220–165 ppm	165–110 ppm	110–45 ppm	45–0 ppm
WF	7.90	12.15	22.05	57.90
MWF	10.38	11.64	14.11	63.88

## Data Availability

Data are available under reasonable request.

## Supplementary Materials

The following supporting information can be downloaded at: https://www.mdpi.com/article/10.3390/toxics12050356/s1, Figure S1: SEM-EDS images of hydrochar WF and MWF; Figure S2: SEM-EDS images of Cd^2+^ and Pb^2+^ adsorbed by hydrochar WF and MWF; Figure S3: FTIR spectra before and after adsorption of Cd^2+^ and Pb^2+^ by hydrochar WF (A) and MWF (B); Figure S4: XRD patterns of hydrochar WF (A) and MWF (B) before and after adsorption of Cd^2+^ and Pb^2+^; Figure S5: The XPS spectra of O1s (A, B), N1s (C, D) and S2p (E, F) of hydrochar WF and MWF before and after adsorption of Cd^2+^ and Pb^2+^; Figure S6: The XPS spectra of Mg1s (A, B) and C1s (C, D) of hydrochar WF and MWF before and after adsorption of Cd^2+^ and Pb^2+^; Figure S7: The C1s (A, B) and N1s (C, D) high-resolution XPS spectra of hydrochar WF (A, C) and MWF (B, D) before and after adsorption of Cd^2+^ and Pb^2+^; Figure S8: Growth of bok choy under different fertilization treatments; Table S1: The element content of chicken feathers (%); Table S2: Nutrient content and basic physicochemical properties of soil tested; Table S3: Fertilizer application method for the bok choy potting test; Table S4: Sulfur content of pyrolysis char and hydrochar in different studies; Table S5: Carbon stability and carbon loss rate of hydrochar determined by H_2_O_2_ oxidation (%); Table S6: Properties and nutrient composition of different organic water-soluble fertilizers; Table S7: Effects of different fertilization treatments on yield indexes of bok choy. References [68,69] are cited in the supplementary materials.
