# Supplementary material for: Adsorption of Cadmium and Lead Capacity and Environmental Stability of Magnesium-Modified High-Sulfur Hydrochar: Greenly Utilizing Chicken Feather
Source: Toxics. 2024 May 11;12(5):356. doi: 10.3390/toxics12050356 (PMC11126130; doi:10.3390/toxics12050356)
Supplement: Supplementary file 1 [file toxics-12-00356-s001.zip › toxics-2968805-supplementary.pdf]

# Supplementary Material

# Adsorption of Cadmium and Lead Capacity and Environmental Stability of Magnesium-Modified High-Sulfur Hydrochar: Greenly Utilizing Chicken Feather

**Weiqi Deng [1,2,3], Xubin Kuang [1,2] , Zhaoxin Xu [1,2] , Deyun Li [4], Yongtao Li [1,2] and Yulong Zhang [1,2,3,*]**

[1] Key Laboratory of Arable Land Conservation (South China), Ministry of Agriculture, College of Natural Resources and Environment, South China Agricultural University, Guangzhou, 510642, China; deng79141@163.com (W.D.); 15918625625@163.com (X.K.); xuzhaoxin0643@163.com (Z.X.); yongtao@scau.edu.cn (Y.L.); yulongzhang@scau.edu.cn (Y.Z.)

[2] College of Natural Resources and Environment, Joint Institute for Environmental Research & Education, South China Agricultural University, Guangzhou 510642, China

3 WENS Foodstuff Grp Co Ltd, Yunfu 527400, China

4 School of Environmental Science and Engineering, Shaanxi University of Science & Technology, Xi'an 710021, China; deyun_94@163.com (D.L.)

* Correspondence: yulongzhang@scau.edu.cn

Table S1.The element content of chicken feathers (%)

| Elemental context | C | H | N | S |
|---|---|---|---|---|
| Chicken feather | 39.88 | 6.04 | 12.07 | 4.02 |

Table S2. Nutrient content and basic physicochemical properties of soil tested

| Index | Unit | Context |
|---|---|---|
| pH | / | 6.34 ± 0.15 |
| Total nitrogen | $g·kg^{-1}$ | 1.69 ± 0.01 |
| Total phosphorus | | 0.66 ± 0.10 |
| Total potassium | | 20.61 ± 0.05 |
| Soil organic matter | | 33.34 ± 0.04 |
| Alkali-hydrolyzable nitrogen | $mg·kg^{-1}$ | 153.00 ± 1.04 |
| Available phosphorus | | 13.23 ± 0.17 |
| Readily available potassium | | 68.60 ± 0.26 |
| Cation exchange capacity | $cmol·kg^{-1}$ | 14.20 ± 0.06 |
| Exchangeable calcium | | 2483.04 ± 1.96 |
| Exchangeable magnesium | | 324.395 ± 2.04 |

Table S3. Fertilizer application method for the bok choy potting test

| | Base fertilizer | First topdressing | Second topdressing | Third topdressing |
|---|---|---|---|---|
| Fertilizer proportion | 40% | 15% | 30% | 15% |
| Fertilizer time | Before transplanting 7 d | After transplanting 7 d | After transplanting 14 d | After transplanting 21 d |

Table S4. Sulfur content of biochar and hydrochar in different studies

| Sulfur context (%) | Pyrolysis process | Reaction temperature (℃) | Raw material | Reference |
|---|---|---|---|---|
| 0.88 | Slow pyrolysis | 450 | Chicken feather | [22] |
| 0.96 | Slow pyrolysis | 450 | Chicken feather | [22] |
| 0.32 | Slow pyrolysis | 450 | Chicken feather | [23] |
| ~0.37 | Slow pyrolysis | 400 | Sewage | [68] |
| 0.02 | Slow pyrolysis | 450 | Corn straw | [69] |
| 1.88 | Hydrothermal carbonization | 150 | Chicken feather | [26] |
| 0.38 | Hydrothermal carbonization | 200 | Rice Straw | [40] |
| 0.31 | Hydrothermal carbonization | 200 | Swine Manure | [40] |
| 3.336 | Hydrothermal carbonization | 180 | Chicken feather | This work |
| 3.683 | Hydrothermal carbonization | 180 | Chicken feather | This work |

Table S5. Carbon stability and carbon loss rate of hydrochar determined by $H_2O_2$ oxidation (%)

| | WF | MWF |
|---|---|---|
| Carbon stability | 58.16 ± 0.8a | 59.42 ± 0.3a |
| Carbon loss rate | 41.84 ± 0.8a | 40.58 ± 0.3a |

Different lowercase letters indicate significant differences between processes ($p < 0.05$).

Table S6. Properties and nutrient composition of different organic water-soluble fertilizers

| | WF | MWF | WFNPK | MWFNPK |
|---|---|---|---|---|
| pH（1：200） | 8.219 ± 0.074 | 7.422 ± 0.065 | 6.360 ± 0.091 | 5.627 ± 0.038 |
| Organic matter ($g\cdot L^{-1}$) | 49.68 ± 0.04 | 53.84 ± 0.61 | 49.68 ± 0.04 | 53.84 ± 0.61 |
| TN ($g\cdot L^{-1}$) | 9.84 ± 0.42 | 7.37 ± 0.21 | 82.48 | 82.48 |
| TP ($g\cdot L^{-1}$) | 0.04 ± 0.00 | 0.12 ± 0.00 | 13.57 | 13.57 |
| TK ($g\cdot L^{-1}$) | 0.02 ± 0.00 | 0.02 ± 0.00 | 44.22 | 44.22 |
| Mg ($g\cdot L^{-1}$) | 0.01 ± 0.00 | 22.69 ± 0.07 | 0.01 ± 0.00 | 22.69 ± 0.07 |

Table S7 Effects of different fertilization treatments on yield indexes of bok choy

| Treatment | CK | NPK | WF | WFNPK | MWFNPK |
|---|---|---|---|---|---|
| Ground fresh weight (g) | 27.71 ± 1.75a | 33.39 ± 2.36b | 32.29 ± 2.85ab | 35.33 ± 2.07bc | 39.04 ± 2.86c |
| Ground dry weight (g) | 1.45 ± 0.20a | 1.63 ± 0.22ab | 1.71 ± 0.28ab | 1.77 ± 0.21ab | 2.09 ± 0.10b |
| Leaf length (cm) | 19.88 ± 2.78a | 20.91 ± 2.24ab | 20.21 ± 1.48ab | 21.79 ± 2.33bc | 22.70 ± 1.21c |
| Leaf width (cm) | 8.18 ± 1.01a | 8.76 ± 1.06ab | 8.70 ± 0.94ab | 8.46 ± 1.11ab | 9.27 ± 1.32b |
| Leaf number | 6.11 ± 0.57a | 6.44 ± 0.68ab | 6.89 ± 0.57bc | 7.00 ± 0.94c | 6.78 ± 0.79bc |
| Plant height (cm) | 21.2 ± 3.04a | 21.90 ± 2.41a | 21.51 ± 1.38a | 22.60 ± 2.24ab | 23.60 ± 1.45b |
| Root length (cm) | 6.40 ± 0.36a | 6.58 ± 1.38a | 7.00 ± 0.80a | 7.37 ± 0.45a | 7.33 ± 1.86a |

Different lowercase letters indicate significant differences between processes ($p < 0.05$)

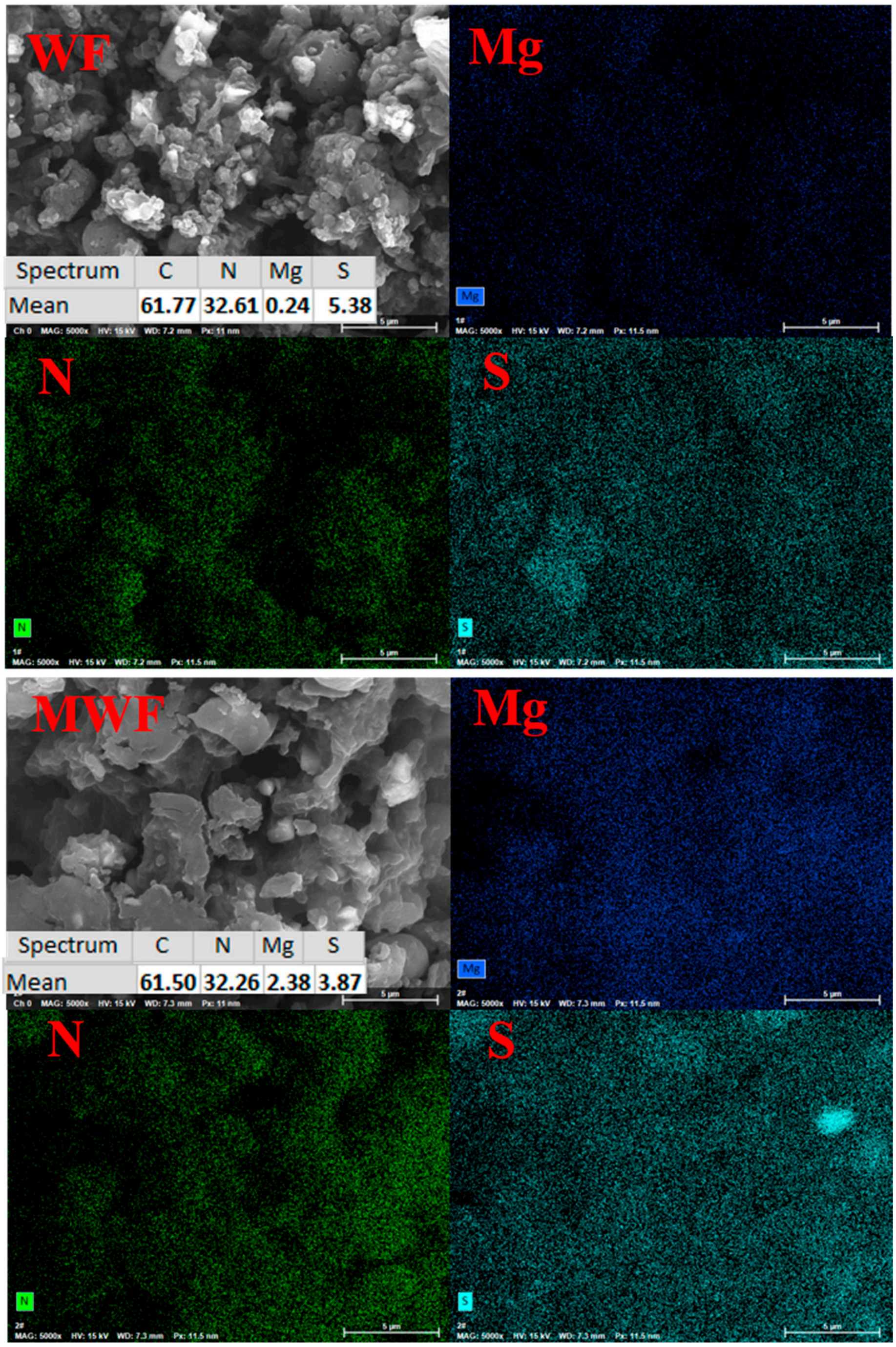


Figure S1. SEM-EDS images of hydrochar WF and MWF

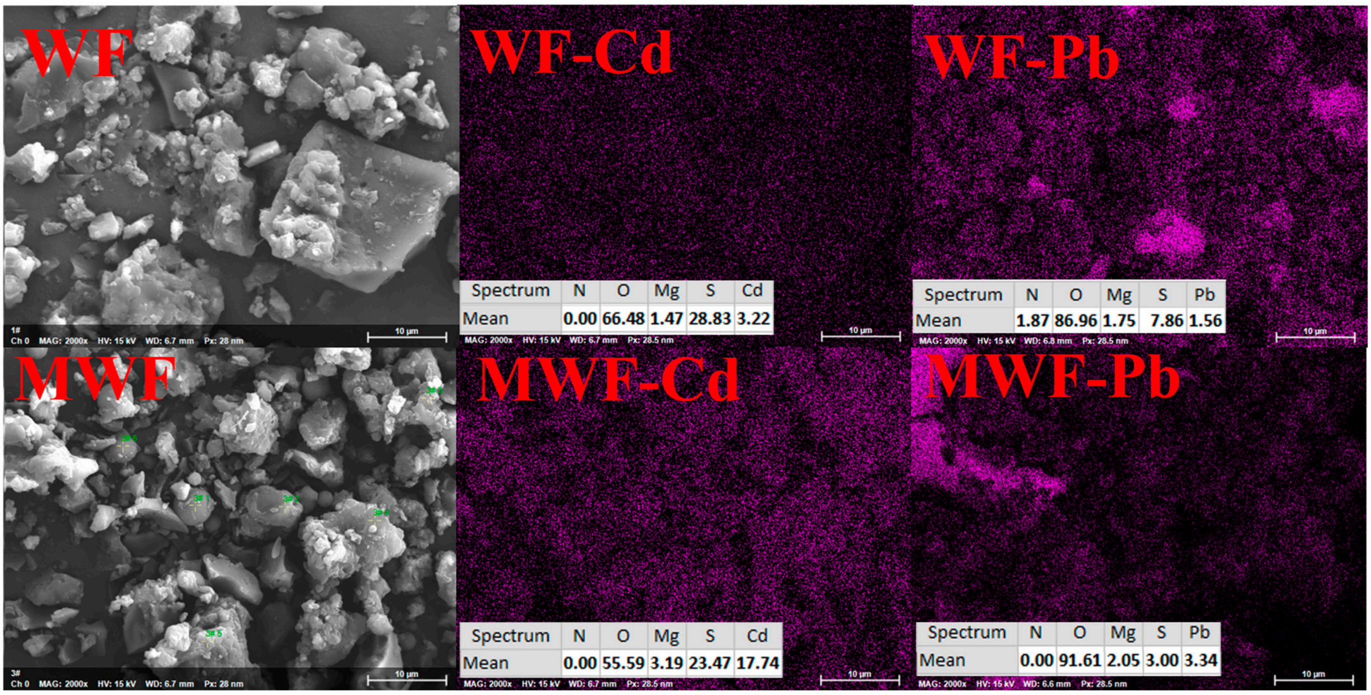


Figure S2. SEM-EDS images of $Cd^{2+}$ and $Pb^{2+}$ adsorbed by hydrochar WF and MWF

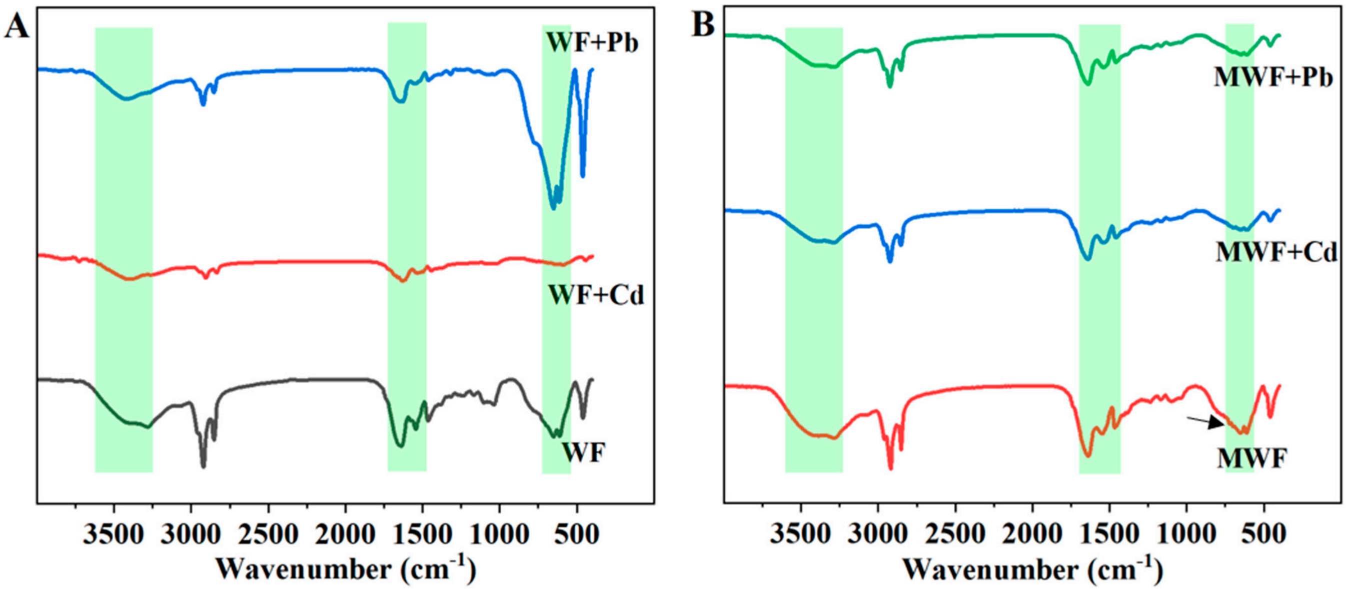


Figure S3. FTIR spectra before and after adsorption of $Cd^{2+}$ and $Pb^{2+}$ by hydrochar WF (A) and MWF (B)

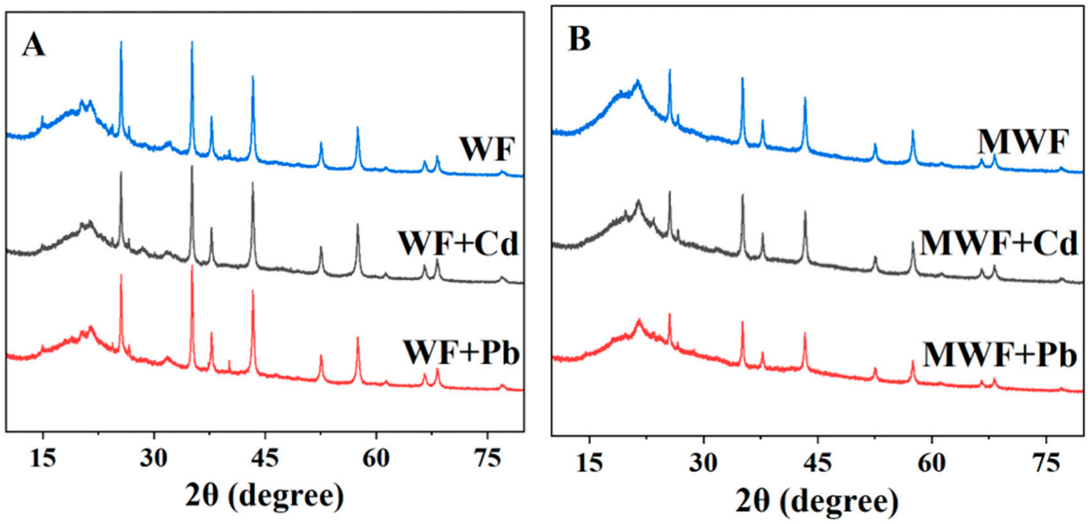


Figure S4. XRD patterns of hydrochar WF (A) and MWF (B) before and after adsorption of $Cd^{2+}$ and $Pb^{2+}$

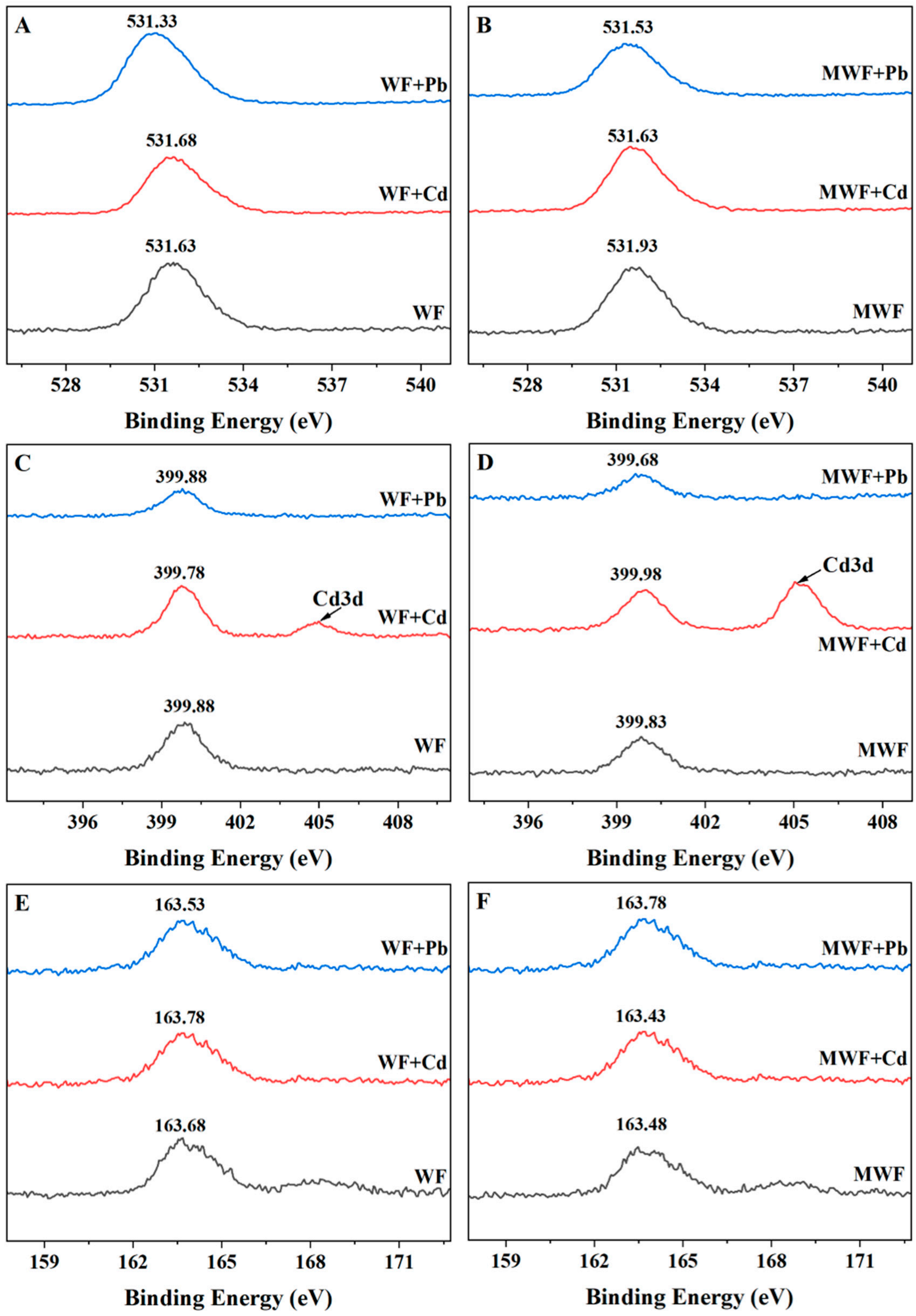


Figure S5. The XPS spectra of O1s (A, B), N1s (C, D) and S2p (E, F) of hydrochar WF and MWF before and after adsorption of $Cd^{2+}$ and $Pb^{2+}$

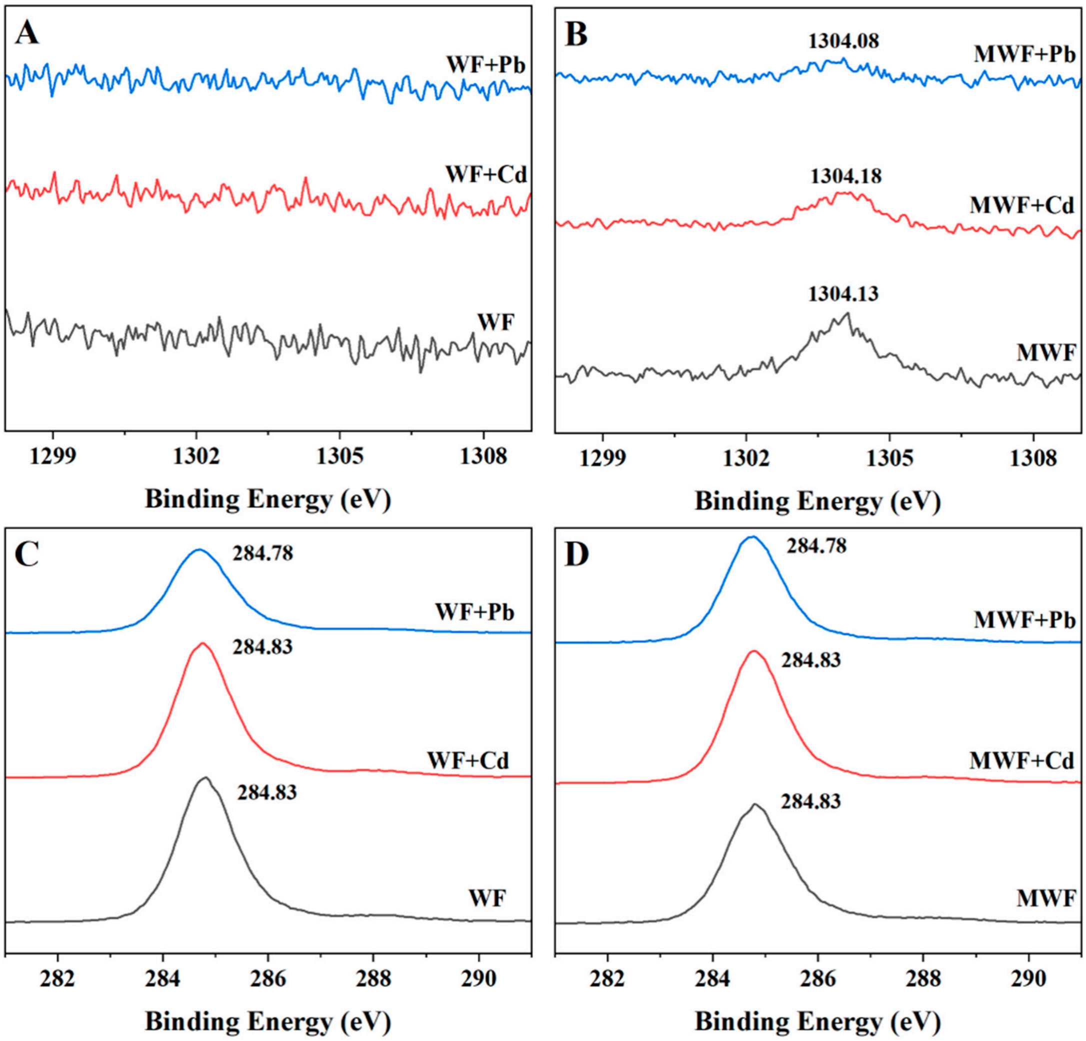


Figure S6. The XPS spectra of Mg1s (A, B) and C1s (C, D) of hydrochar WF and MWF before and after adsorption of $Cd^{2+}$ and $Pb^{2+}$

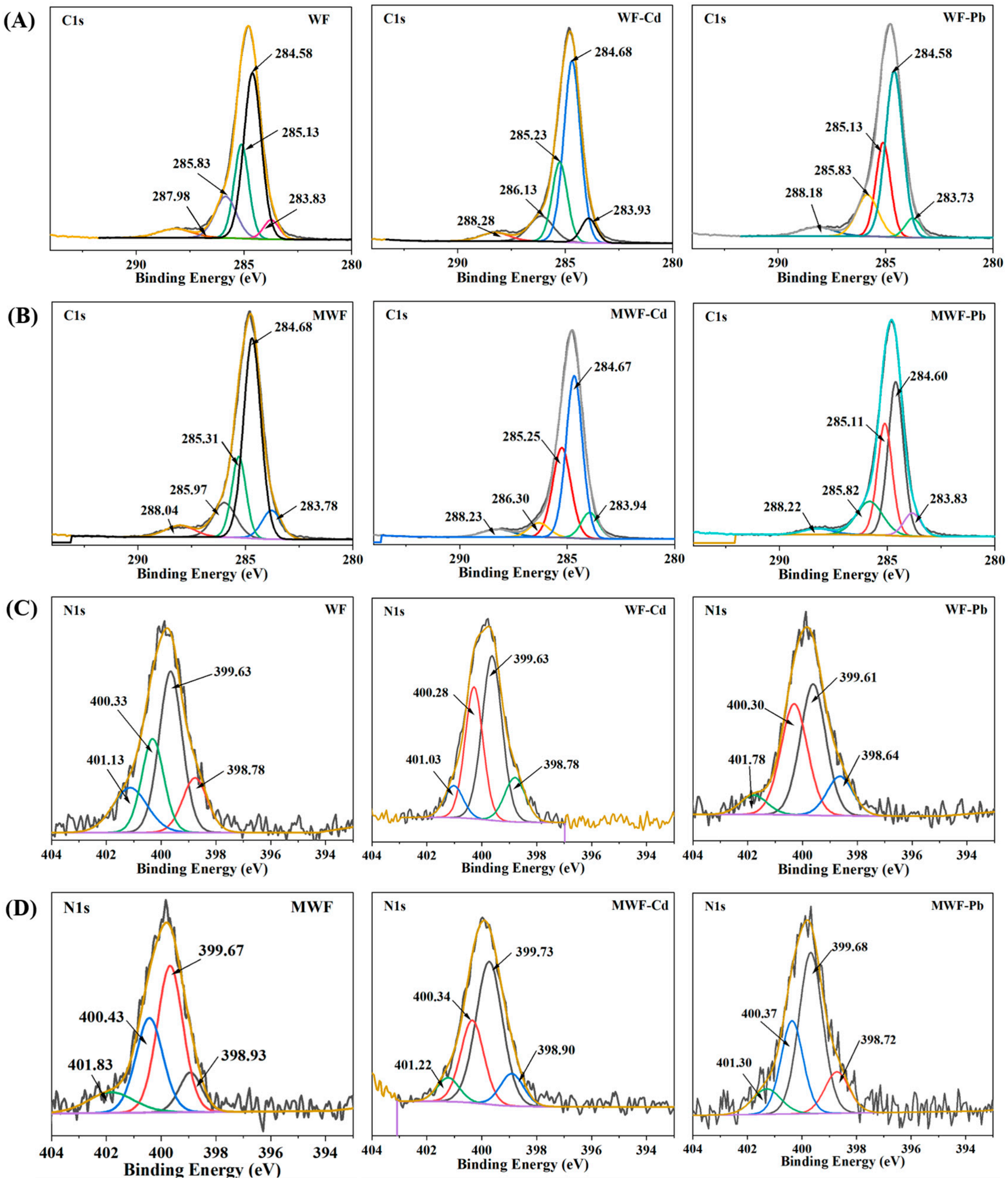


Figure S7. The C1s (A, B) and N1s (C, D) high-resolution XPS spectra of hydrochar WF (A, C) and MWF (B,D) before and after adsorption of $Cd^{2+}$ and $Pb^{2+}$

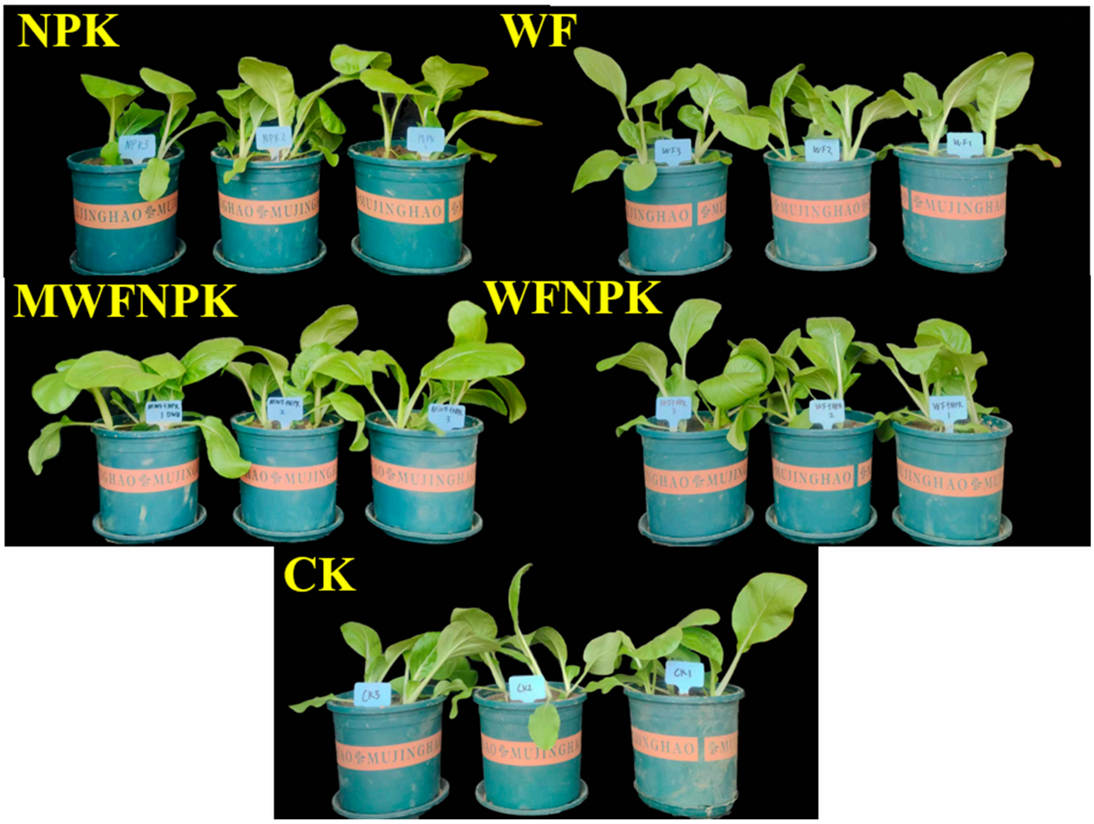


Figure S8. Growth of bok choy under different fertilization treatments
